# Ultrasound-Guided Dextrose Injection and Hydrodissection at the Soleus Fibular Enthesis: A Technical Report

**DOI:** 10.7759/cureus.117855

**Published:** 2026-10-13

**Authors:** Yonghyun Yoon, Hyeongjik Kim, Dongyeun Sung, Jonghyeok Lee, Seungbeom Kim, Youngmo Kim, Teinny Suryadi, Anwar Suhaimi, King Hei Stanley Lam

**Affiliations:** 1 Orthopedics, International Academy of Musculoskeletal Medicine, Hong Kong, HKG; 2 Orthopedics, International Academy of Regenerative Medicine, Seoul, KOR; 3 Orthopedic Surgery, Hallym University Kangnam Sacred Heart Hospital, Seoul, KOR; 4 Orthopedic Surgery, Incheon Terminal Orthopedic Surgery Clinic, Incheon, KOR; 5 Orthopedic Surgery, International Academy of Regenerative Medicine, Seoul, KOR; 6 Neurosurgery, Himchannamu Neurosurgery Clinic, Daegu, KOR; 7 Neurosurgery, Bareun Neurosurgery Clinic, Cheongju-si, KOR; 8 Pain Medicine, Miso Pain Clinic, Suwon, KOR; 9 Orthopedics, Incheon Terminal Orthopedic Surgery Clinic, Incheon, KOR; 10 Physical Medicine and Rehabilitation, Medistra Hospital, Central Jakarta, IDN; 11 Physical Medicine and Rehabilitation, Synergy Clinic, Jakarta, IDN; 12 Physical Medicine and Rehabilitation, Hermina Podomoro Hospital, Jakarta, IDN; 13 Rehabilitation Medicine, University Malaya Medical Centre, Kuala Lumpur, MYS; 14 Rehabilitation Medicine, Universiti Malaya, Kuala Lumpur, MYS; 15 Clinical Research, International Academy of Regenerative Medicine, Seoul, KOR; 16 The Board of Clinical Research, Indonesia Malaysia Regenerative Institute, Jakarta, IDN; 17 The Board of Clinical Research, The International Association of Musculoskeletal Medicine, Kowloon, HKG; 18 Faculty of Medicine, The Chinese University of Hong Kong, New Territories, HKG; 19 Faculty of Medicine, The University of Hong Kong, Hong Kong, HKG; 20 The Board of Clinical Research, The Hong Kong Institute of Musculoskeletal Medicine, Kowloon, HKG

**Keywords:** 5% dextrose in water, bone stress injury, hydrodissection, periosteal reaction, prolotherapy, proximal fibula, soleus fibular enthesis

## Abstract

This technical report describes an ultrasound-guided lesion-directed procedure at the soleus fibular enthesis in a patient with a clinically diagnosed proximal fibular bone stress injury and distal sensory symptoms. A 68-year-old female presented with persistent right Achilles-region pain, dorsal foot paresthesia, focal proximal fibular tenderness, and no foot drop; ankle eversion was pain-limited to Medical Research Council grade 4/5. Radiographs showed periosteal reaction at the symptomatic proximal fibula, interpreted clinically as a bone stress injury at the soleus attachment. With the patient prone, a 23-gauge, 6-cm needle was advanced in-plane from caudal to cranial toward the periosteal stress region. Aspiration was performed immediately before injection as a blood-return safety check. Five percent dextrose in water (D5W) was delivered to the symptomatic attachment region. The same needle was then redirected medially within the attachment-related treatment field, and additional D5W was used to produce visible separation of the adjacent soft-tissue plane. The common peroneal nerve (CPN) was neither contacted nor directly hydrodissected. A total of 30 mL of D5W was used per session for six sessions at two-week intervals. Reported global symptom improvement was approximately 50% after the first session and 80% after the sixth; final follow-up occurred three months after treatment completion, with no reported adverse events. The procedure therefore combined lesion-directed dextrose delivery with attachment-adjacent hydrodissection within one anatomical treatment region. The observed response does not establish treatment efficacy, a CPN-specific mechanism, enthesis regeneration, or fracture healing.

## Introduction

Bone stress injury (BSI) encompasses stress reactions and stress fractures and may arise from repetitive loading of otherwise normal bone or from physiologic loading of bone with impaired mechanical competence [1]. Proximal fibular stress fracture is uncommon, and focal tenderness can help localize a symptomatic lesion [2]. The soleus has a substantial proximal fibular attachment, including a tendinous component at the posterior fibular head and aponeurotic fibers extending along the fibula [3]. This anatomy provides a structural basis for considering the attachment region when periosteal stress change and focal tenderness coincide at the proximal fibula.

Distal sensory symptoms in this region may prompt assessment of the common peroneal nerve (CPN), and direct CPN hydrodissection has been described for compressive neuropathy [4]. However, symptom distribution alone does not establish the nerve as the procedural target. Hydrodissection is characterized by visible fluid-mediated separation of an intended tissue interface, whereas dextrose prolotherapy involves target-directed dextrose delivery to a symptomatic connective-tissue structure; injectate concentration alone does not determine the procedural category [5,6].

A similar target-selection dilemma may arise when a structurally concordant lesion coexists with distal nerve-related symptoms in another anatomical region. In deep gluteal syndrome (DGS), muscle-tendon, entheseal, or ligamentous lesions may serve as primary structural pain generators with secondary nerve-related symptoms [7]. Lesion-directed dextrose treatment has been reported for sacrospinous-ligament calcification and piriformis enthesopathy associated with DGS [8,9]. These reports provide a clinical analogy for lesion-directed target selection rather than evidence of an identical pathophysiologic mechanism. Five percent dextrose in water (D5W) was selected because a single injectate could be used for both attachment-directed dextrose delivery and subsequent fluid-mediated tissue-plane separation; this was a procedural choice and does not imply superiority over saline or local anesthetic or establish a specific pharmacologic or regenerative mechanism. The technical contribution of the present report is a reproducible lesion-directed approach combining these two procedural functions within a single soleus fibular enthesis treatment region, without direct CPN intervention.

## Technical report

Clinical presentation and target selection

A 68-year-old female with rheumatoid arthritis treated with methotrexate and intermittent corticosteroids as needed, hypertension, and dyslipidemia presented with several months of persistent right heel pain and dorsal foot paresthesia. She had no history of bisphosphonate use, and erythrocyte sedimentation rate and C-reactive protein data were unavailable for this episode. Walking for more than approximately 10 minutes reproducibly provoked pain around the Achilles tendon and discomfort extending distally from the fibular-neck region toward the heel. Examination showed focal proximal fibular tenderness. There was no foot drop. Ankle dorsiflexion and toe extension were preserved, whereas ankle eversion strength was Medical Research Council grade 4/5 and limited by pain [10]. Despite the reported dorsal foot paresthesia, bedside sensory examination was intact. Achilles tendon ultrasonography and electrodiagnostic or other nerve-specific physiological testing were not performed. Accordingly, the available clinical assessment did not establish a discrete CPN neuropathy.

Radiographs demonstrated periosteal reaction along the proximal right fibula near the neck-shaft region (Figure 1). On clinical-radiographic correlation, the lesion was interpreted as a proximal fibular BSI at the soleus fibular attachment, within the clinical spectrum that includes stress reaction and stress fracture [1]. The soleus fibular enthesis and its immediately adjacent periosteal-soft-tissue interface were treated as one anatomical target region. Secondary local irritation involving the CPN was considered a possible explanation for the distal sensory symptoms, but a nerve-specific mechanism was not established.

**Figure 1:**
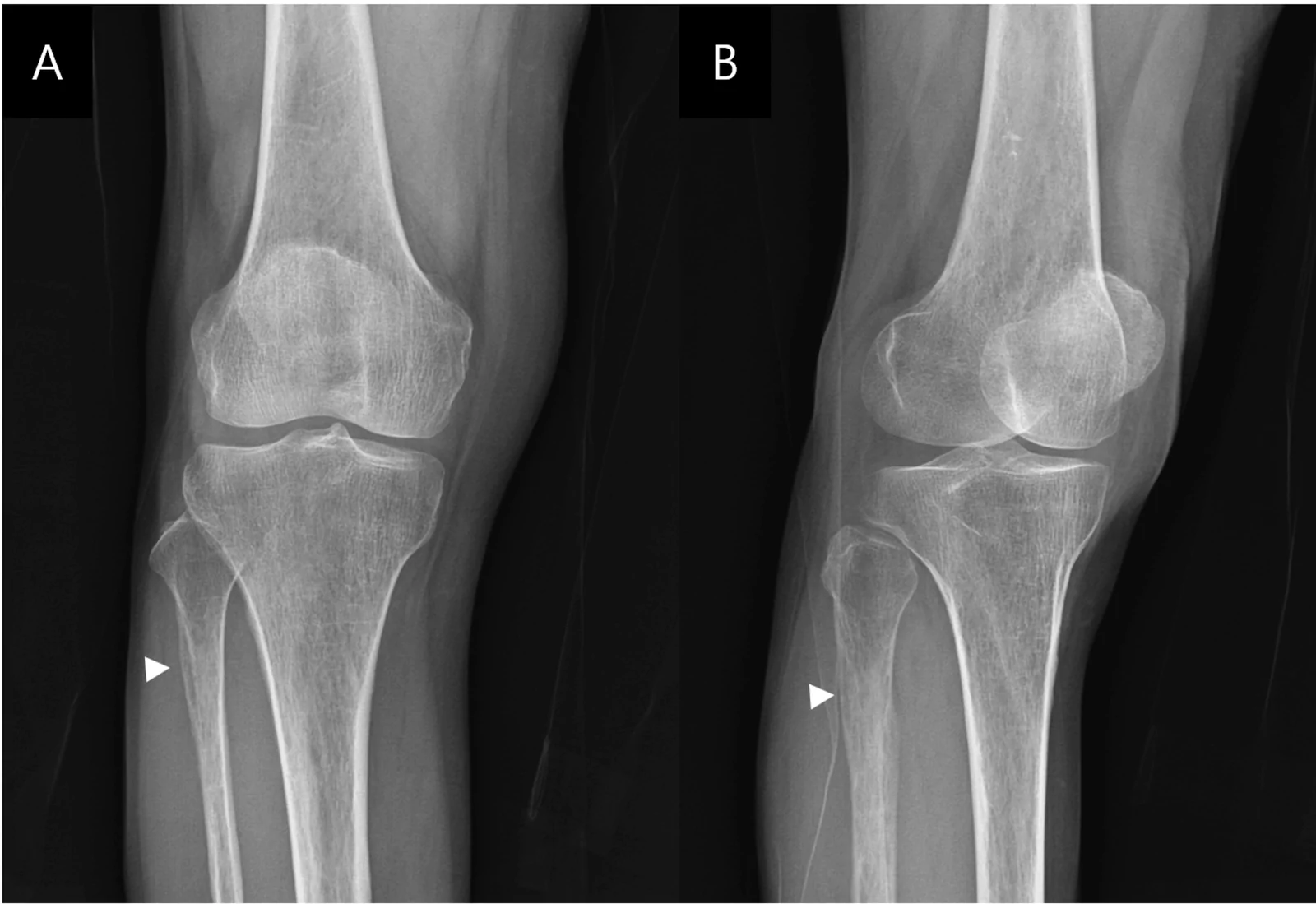
Proximal fibular periosteal reaction at the symptomatic site. Two radiographic views of the right knee and proximal leg (A, B) show focal periosteal reaction at the proximal fibular neck-shaft region (arrowheads). Together with focal tenderness and the clinical presentation, this finding was interpreted as a bone stress injury involving the soleus fibular attachment.

Positioning and sonographic landmarks

Ultrasound was performed with the patient prone using an X-CUBE 90 with Elite system (ALPINION Medical Systems Co., Ltd., Seoul, Republic of Korea) and a linear transducer. The fibular head was identified first, followed by the neck and proximal shaft. A longitudinal view provided orientation to the fibular contour and the overlying lateral gastrocnemius (Figure 2). The known proximal fibular origin of the soleus was used to relate the radiographic lesion to the attachment region [3]. In the displayed longitudinal image, caudal is on the left and cranial on the right.

**Figure 2:**
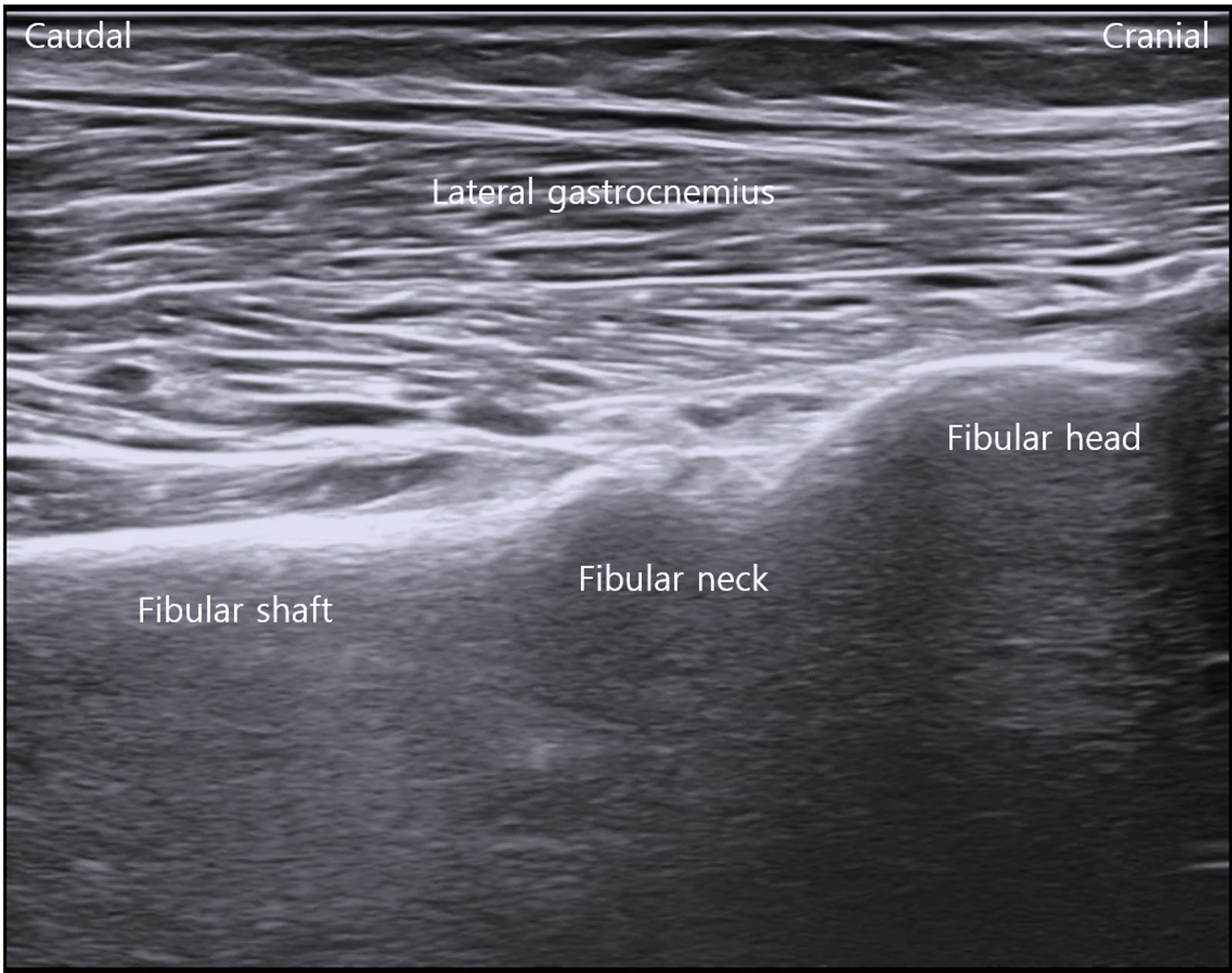
Longitudinal ultrasound landmarks of the proximal fibula. The fibular shaft, neck, and head are labeled, with the lateral gastrocnemius superficial to the fibular contour. Caudal is displayed on the left and cranial on the right. This image provides regional orientation before target-directed intervention.

Two-plane scanning sequence

In the axial orientation, the transducer was translated from cranial to caudal. In the sagittal orientation, it was translated from lateral to medial. These complementary movements are illustrated in Figure 3 and Videos 1, 2. The scanning directions are distinct from the caudal-to-cranial needle trajectory used during treatment.

**Figure 3:**
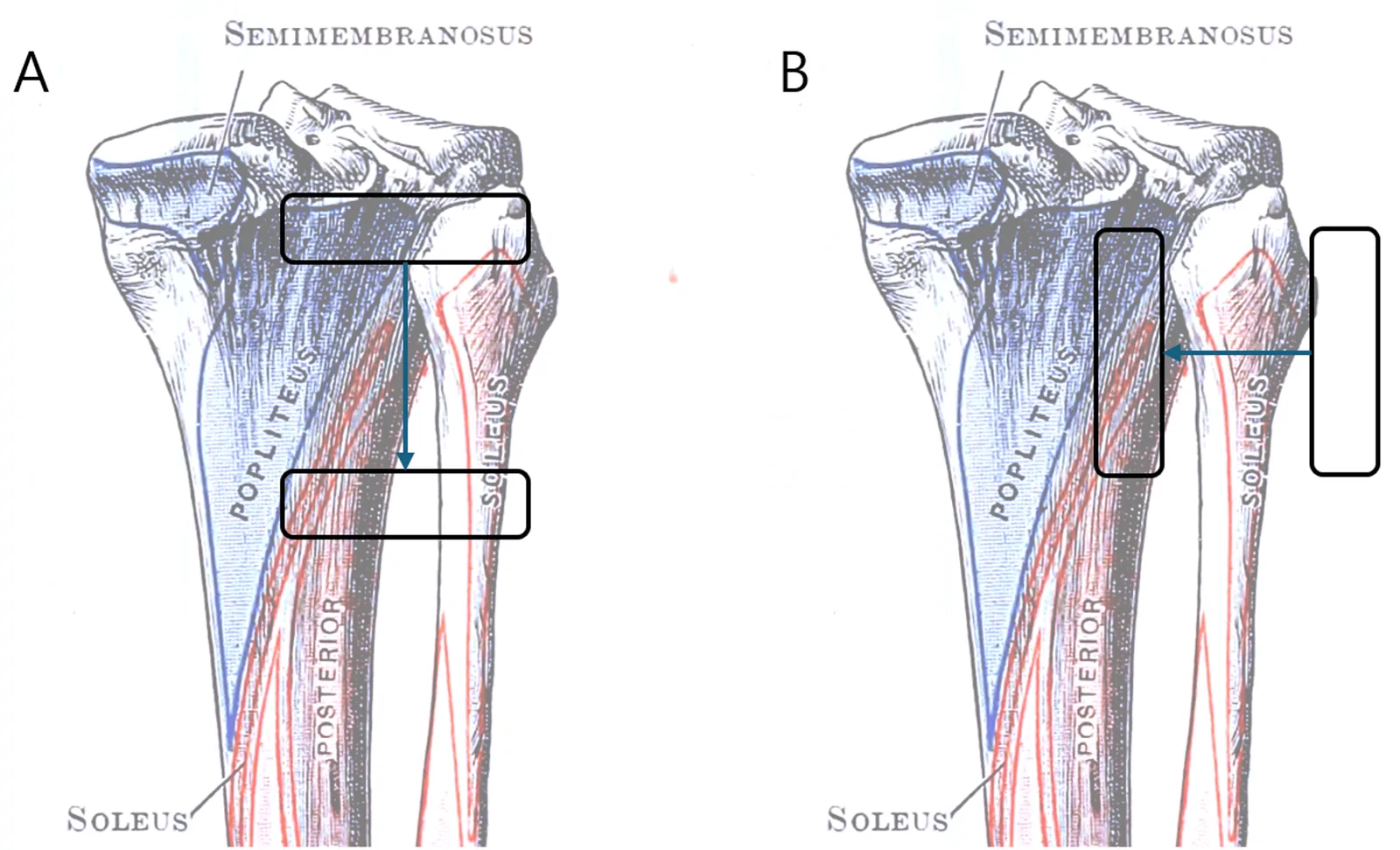
Transducer orientation and scanning directions. (A) Axial scanning progresses from cranial to caudal. (B) Sagittal scanning progresses from lateral to medial. White rectangles indicate starting positions and black rectangles indicate subsequent positions. Blue arrows indicate transducer translation, not needle advancement. The anatomical image was adapted from Figure 251 of Cunningham's Text-book of Anatomy (1914) [11]. The figure is in the public domain.

**Video 1:** Cranial-to-caudal axial ultrasound scanning. With the patient prone, the transducer is maintained in an axial orientation and moved from cranial to caudal through the proximal calf. The sequence surveys regional muscular, osseous, and neurovascular relationships before treatment.

**Video 2:** Lateral-to-medial sagittal ultrasound scanning. With the patient prone, the transducer is maintained in a sagittal orientation and moved from the lateral proximal fibular region toward the medial proximal leg tissue planes. Identification of the CPN during the diagnostic survey did not define it as the subsequent injection target. CPN, common peroneal nerve.

D5W injection and attachment-adjacent hydrodissection

The patient remained prone, and the transducer was positioned longitudinally over the symptomatic proximal fibula. A 23-gauge, 6-cm needle was advanced in-plane from caudal to cranial toward the periosteal stress region at the soleus fibular attachment (Figure 4). The intended treatment field remained centered on the same attachment-related region throughout the procedure.

**Figure 4:**
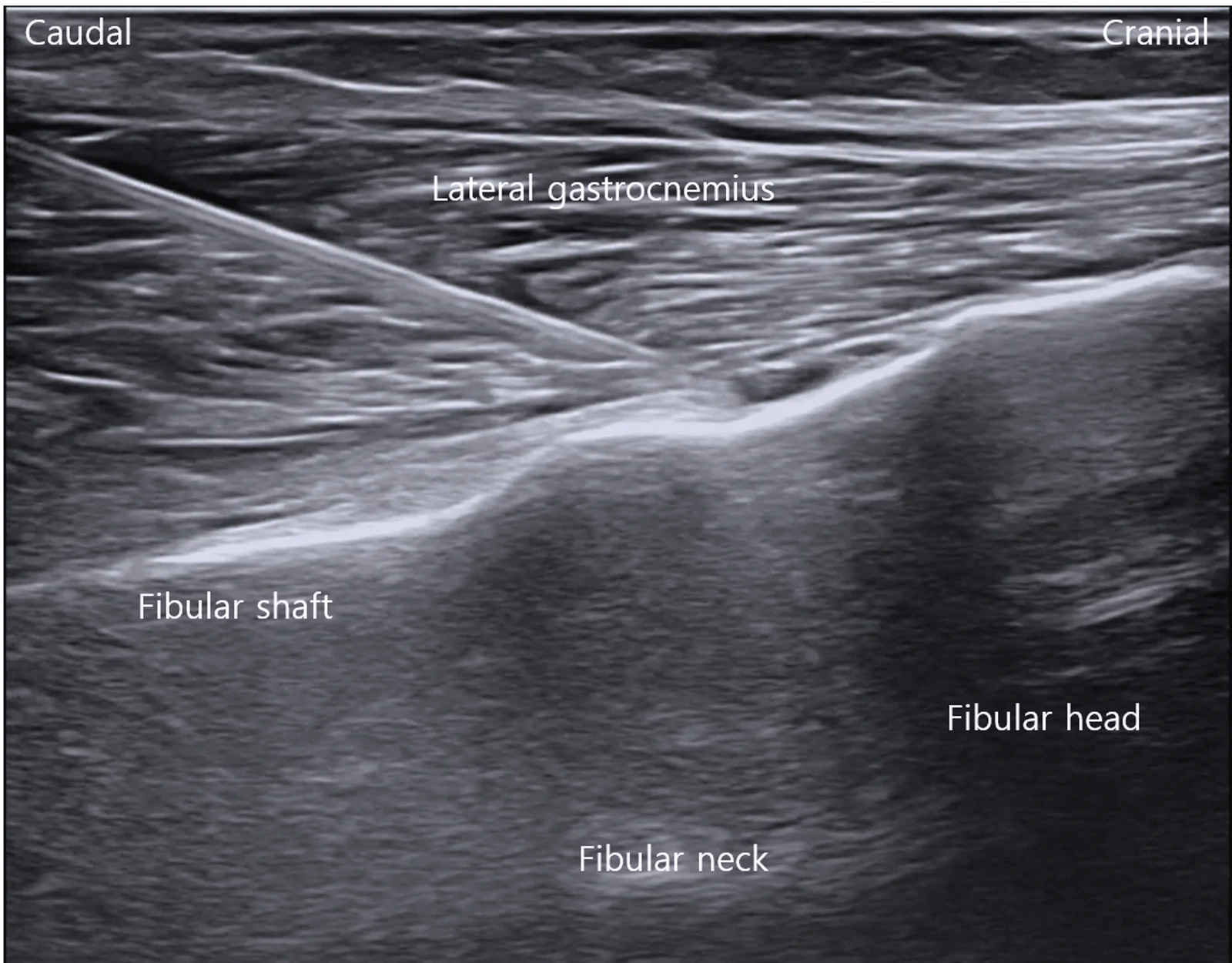
Initial caudal-to-cranial in-plane approach to the proximal fibular periosteal interface. The needle enters from the caudal side on the left and advances toward the symptomatic proximal fibular attachment region on the right. The fibular head, neck, and shaft, and the lateral gastrocnemius provide regional orientation. This still image depicts the initial approach; the subsequent medial redirection and attachment-adjacent hydrodissection are described in the procedural text and Video 3.

**Video 3:** Ultrasound-guided hydrodissection at the proximal fibular periosteal-enthesis region. The recording illustrates the initial in-plane caudal-to-cranial approach and local fluid spread. The white arrowhead indicates the periosteal reaction at the proximal fibula. The complete procedure included aspiration before injection and subsequent medial redirection toward the soleus attachment, using a 23-gauge, 6-cm needle and 30 mL of D5W in total per session. Not every step of the complete treatment is separately shown in this short clip. D5W, 5% dextrose in water.

Aspiration was performed immediately before injection to check for blood return. D5W was first delivered under ultrasound guidance to the symptomatic soleus fibular attachment/periosteal stress region as lesion-directed dextrose delivery. The same needle was then redirected medially within the attachment-related treatment field, and additional D5W was used to open the adjacent soft-tissue plane under real-time ultrasound guidance. A total of 30 mL of D5W was used per session; the volume allocated to each procedural phase was not separately recorded. The CPN was not contacted, injected, deliberately surrounded with injectate, or directly hydrodissected.

Real-time imaging demonstrated local fluid spread and separation of the intended attachment-adjacent soft-tissue plane (Video 3). These sequential steps occurred within one anatomical treatment region but served two procedural functions: lesion-directed dextrose delivery and fluid-mediated tissue-plane separation. In this report, hydrodissection refers to separation of the attachment-adjacent soft-tissue interface, not detachment of the enthesis from bone. Circumferential fluid spread around the CPN was not a procedural endpoint.

Rationale for D5W and procedural classification

D5W was selected because a single injectate could serve both procedural functions within the same treatment region. Other injectates, including saline or local anesthetic, can also facilitate mechanical tissue-plane separation [5,6], but they were not evaluated in this case, and no comparative claim is made. D5W is widely used in ultrasound-guided hydrodissection, particularly in perineural applications, while its proposed analgesic mechanisms remain incompletely defined [5,6]. Here, the rationale was primarily procedural: the intervention required an injectate suitable for fluid-mediated tissue-plane separation while also permitting low-concentration dextrose delivery to the symptomatic connective-tissue attachment without changing injectates. This description does not imply that the procedure was conventional high-concentration prolotherapy or that a pharmacologic regenerative effect was demonstrated.

Terminology was assigned according to the anatomical target and immediate procedural endpoint rather than dextrose concentration alone. Five percent dextrose has been studied as low-dose prolotherapy when deliberately delivered to an enthesis [12]. Accordingly, the attachment-directed phase in this case is consistent with a low-dose dextrose prolotherapy component, whereas the phase in which visible fluid-mediated tissue-plane separation occurred fulfills the operational definition of hydrodissection [6]. Hydrodissection has also been studied in non-perineural myofascial tissue planes [13]. These two procedural functions occurred within one attachment-related treatment region and should not be interpreted as two separate anatomical targets. The term enthesis-directed describes the anatomical target and does not establish chronic degenerative enthesopathy as the diagnosis; the radiographic lesion was interpreted primarily as BSI at the attachment.

Treatment schedule and clinical response

Six sessions were performed at two-week intervals, each using 30 mL of D5W across the procedure. Reported global symptom improvement was approximately 50% immediately after the first session and 80% after the sixth session. These percentages were not converted into numerical rating scale or visual analog scale scores and were not recorded separately for pain and paresthesia. The baseline walking-provocation threshold of approximately 10 minutes was not reassessed using a standardized protocol at treatment completion or at the final follow-up and therefore cannot be reported as a comparative functional outcome. Final follow-up occurred three months after completion of treatment; no additional numerical symptom estimate was recorded at that visit. Adverse events were assessed by direct patient questioning and clinical assessment at each treatment visit and at the final follow-up; no treatment-related adverse event was identified.

Ethics and consent

In accordance with the treating institution's policy for single-patient case reports, institutional review board review was not required. The patient provided informed consent for publication of the clinical information and media.

## Discussion

Target selection and symptom distribution

Treatment was directed to a single anatomical region centered on the clinically concordant soleus fibular enthesis, periosteal stress lesion, and immediately adjacent soft-tissue interface. The CPN was not directly hydrodissected. A local attachment-related stress lesion with possible secondary neural irritation was considered as one explanation for the distal symptoms, but improvement after lesion-directed treatment does not isolate a neural pathway or establish that the CPN caused all sensory complaints.

The soleus originates from the proximal fibula and contributes distally to the Achilles tendon, providing anatomical continuity between the proximal attachment region and the posterior leg [3]. This relationship supports anatomical plausibility but does not establish a specific referred-pain mechanism. Likewise, the absence of direct CPN contact does not exclude indirect effects of regional injectate spread. The present report therefore documents target selection, needle trajectory, and immediate sonographic behavior of the injectate rather than a nerve-specific mechanism of analgesia.

Procedural classification and structural-lesion context

The procedure incorporated two sequential functions within one treatment region. The first phase delivered D5W to the symptomatic connective-tissue attachment; the second produced visible fluid-mediated separation of the adjacent soft-tissue interface. Within the target-based framework, the first phase is consistent with a low-dose dextrose prolotherapy component and the second meets the operational definition of hydrodissection [6]. A randomized trial has used 5% dextrose as low-dose prolotherapy at forearm extensor entheses [12], and hydrodissection has been studied in non-perineural myofascial planes [13]. These studies support the procedural terminology but do not establish efficacy for proximal fibular BSI or validate the 30-mL protocol used here.

The DGS literature provides a clinical analogy for lesion-directed target selection. Muscle-tendon and entheseal abnormalities have been recognized as potential primary structural contributors to DGS [7], and lesion-directed prolotherapy has been reported for sacrospinous-ligament calcification and piriformis enthesopathy associated with sciatic-like symptoms [8,9]. The shared principle is selection of a clinically concordant structural lesion as the treatment target rather than automatic treatment of the adjacent nerve. These reports do not establish mechanistic equivalence between chronic DGS lesions and a proximal fibular BSI.

The distinction between procedural classification and biological inference is important in this case. The use of D5W does not demonstrate a regenerative prolotherapy mechanism, and visible tissue-plane separation does not establish that hydrodissection was responsible for the clinical response. No published evidence directly establishes that D5W accelerates healing of a proximal fibular BSI or regenerates the soleus enthesis.

Technical implications and limitations

The practical contribution of this report is the reproducible sequence of caudal-to-cranial access to the symptomatic proximal fibular attachment region, lesion-directed D5W delivery, and medial needle redirection within the attachment-related treatment field for adjacent tissue-plane separation. No established optimal injectate volume has been defined for this specific indication. Accordingly, the 30-mL total volume and two-week interval describe the protocol used in this patient and should not be interpreted as evidence-derived or optimized dosing. The procedure does not replace evaluation and management of the underlying BSI. Conservative management has produced recovery in reported proximal fibular stress fractures, making natural healing an important competing explanation for improvement [2].

This single-patient technical report has no comparator and cannot determine whether lesion-directed dextrose delivery, the hydrodissection phase, D5W itself, natural history, concurrent care, or their combination accounted for the observed response. The diagnosis was based on clinical-radiographic correlation of focal tenderness and periosteal reaction rather than MRI confirmation or a predefined fracture-healing endpoint. In an older patient with rheumatoid arthritis, periosteal reaction may have a broader differential diagnosis, and the available evaluation cannot fully exclude alternative inflammatory, metabolic, medication-related, or mechanical contributors. The report does not distinguish a fatigue-type from an insufficiency-type bone stress mechanism [1]. Standardized sensory mapping, electrodiagnostic testing, and other nerve-specific physiological endpoints were not available for this report. Age, rheumatoid arthritis, and its treatment may influence bone and soft-tissue biology. Phase-specific injectate volumes were not recorded, walking tolerance was not reassessed using a standardized protocol, and outcomes were based on global percentage improvement rather than separate validated pain, sensory, or functional measures. Prospective studies should document the exact receiving plane, injectate distribution, phase-specific volume, safety, concurrent treatment, validated symptom outcomes, interval assessment of the osseous lesion, and a prespecified differential evaluation.

## Conclusions

This single-patient technical report describes ultrasound-guided D5W delivery and attachment-adjacent hydrodissection at the soleus fibular enthesis in a patient with a clinically diagnosed proximal fibular BSI and distal symptoms. Both procedural functions were performed within one anatomical treatment region, without direct CPN intervention. The observed clinical improvement represents a temporal association and does not establish treatment efficacy, enthesis regeneration, fracture healing, a CPN-specific mechanism, or comparative benefit. The technique warrants further evaluation using standardized clinical outcomes and interval assessment of the osseous lesion.
